# On-Machine Detection of Sub-Microscale Defects in Diamond Tool Grinding during the Manufacturing Process Based on DToolnet

**DOI:** 10.3390/s22072426

**Published:** 2022-03-22

**Authors:** Wen Xue, Chenyang Zhao, Wenpeng Fu, Jianjun Du, Yingxue Yao

**Affiliations:** School of Mechanical Engineering and Automation, Harbin Institute of Technology (Shenzhen), Shenzhen 518055, China; xuewencool@163.com (W.X.); 20s153227@stu.hit.edu.cn (W.F.); jjdu@hit.edu.cn (J.D.); yxyao@hit.edu.cn (Y.Y.)

**Keywords:** tool condition monitoring, diamond tools, small target detection, neural networks

## Abstract

Nowadays, tool condition monitoring (TCM), which can prevent the waste of resources and improve efficiency in the process of machining parts, has developed many mature methods. However, TCM during the production of cutting tools is less studied and has different properties. The scale of the defects in the tool production process is tiny, generally between 10 μm and 100 μm for diamond tools. There are also very few samples with defects produced by the diamond tool grinding process, with only about 600 pictures. Among the many TCM methods, the direct inspection method using machine vision has the advantage of obtaining diamond tool information on-machine at a low cost and with high efficiency, and the method is accurate enough to meet the requirements of this task. Considering the specific, above problems, to analyze the images acquired by the vision system, a neural network model that is suitable for defect detection in diamond tool grinding is proposed, which is named DToolnet. DToolnet is developed by extracting and learning from the small-sample diamond tool features to intuitively and quickly detect defects in their production. The improvement of the feature extraction network, the optimization of the target recognition network, and the adjustment of the parameters during the network training process are performed in DToolnet. The imaging system and related mechanical structures for TCM are also constructed. A series of validation experiments is carried out and the experiment results show that DToolnet can achieve an 89.3 average precision (AP) for the detection of diamond tool defects, which significantly outperforms other classical network models. Lastly, the DToolnet parameters are optimized, improving the accuracy by 4.7%. This research work offers a very feasible and valuable way to achieve TCM in the manufacturing process.

## 1. Introduction

Cutting tools are indispensable for machining, especially in turning, milling, drilling, and other subtractive manufacturing processes. The timely acquisition of tool condition information is very important for production and processing. The cost components of the machined products show that tool wear accounts for 2–30% of the total cost [1], which does not take into account the production of defective products due to excessive tool wear. Production lines that do not incorporate a tool condition monitoring (TCM) system cost more to operate. In terms of production time, the downtime due to accidents caused by tool wear accounts for 20% of the whole production time [2]. Therefore, TCM has always been an important topic and issue of focus in the machining field. TCM generally means the detection of the tool’s condition or the prediction of its service life by collecting a signal from the tool during or after machining, such as the current signal, the power signal, the cutting force signal, the vibration signal, tool surface image information, etc. [3].

Compared with TCM in the process of machining a workpiece, TCM during the manufacturing process of the tool itself is still less studied. For example, the diamond-forming turning tools used in precision or ultra-precision machining still need to be trimmed on the tool grinder to ensure the precision of its cutting edge. In this process, a TCM system is needed to monitor the condition of the diamond tool so as to adjust the machining parameters when defects occur.

With the emergence of new technologies, TCM methods are constantly evolving, and researchers have proposed various methods to obtain as much detailed information about the true condition of the tools as possible. Table 1 summarizes the commonly used TCM methods. From the perspective of measurement and tool contact, TCM methods can be categorized into two types: indirect methods and direct methods [4,5]. In addition, some emerging multi-sensor methods [6,7,8], as well as some big data and artificial intelligence analysis methods, are also the focus of TCM [9].

Regarding the indirect methods, they are popularly used to analyze tool conditions by measuring factors such as the cutting forces or vibrations during machining. However, when the size of the tool is tiny, environmental noise, processing temperature, and chips may interfere with the sensor’s signal acquisition, which makes it difficult to obtain signals that can truly reflect the processing condition. In some of the indirect measures listed in Table 1, such as monitoring tools by measuring the vibration signal of a machine tool’s spindle, there is usually little contact between the tool and the grinding wheel. In this situation, signals such as force, vibration, and power are too weak to be used as the basis for detecting a tool’s condition [29].

The direct methods aim to realize TCM by directly capturing tool-wear images [30] or radioactive results [15]. One of the universal testing methods in the production of diamond tools is to directly observe the cutting edge’s morphology and contour under an atomic force microscope (AFM) [11] or a scanning electron microscope (SEM) [31,32,33], both of which are time-consuming. When the cutting edge has no obvious defects (generally referring to the microchipping of the cutting edge), the cutting tool can be determined as fit for purpose. Hence, a more common tool detection method is the use of optical inspection instruments to directly observe the tool’s condition. However, the common disadvantage of these direct TCM methods is that the tool needs to be reinstalled for reprocessing when microchipping is found, which is time-consuming and causes errors due to the repeated positioning of the tool.

**Figure 1:**
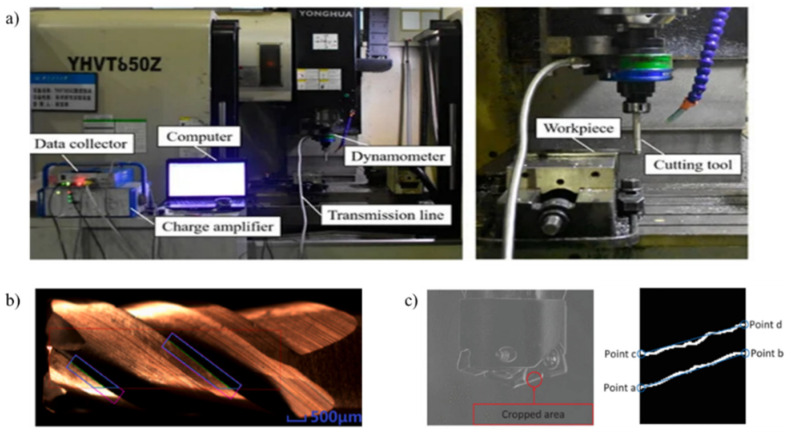
(**a**) Cutting tool on-line monitoring physical platform [4]; (**b**) Tiny-yolo network detection results [37]; (**c**) Contour recognition effect of a milling cutter [2].

Compared with the AFM and SEM methods, optical methods, especially visual detection methods, have been widely used in TCM. Their non-contact measurement capacity makes on-machine measurement possible. Figure 1 shows several methods of applying machine vision inspection in TCM. In Figure 1a, a conventional imaging system experimental platform is shown. TCM using an improved deep learning method is shown in Figure 1b. Figure 1c shows tool-contour extraction using traditional visual inspection methods. Generally, the visual detection methods are promising, not only because they employ a high-information-throughput method to directly obtain the true condition of the tool without touching the tool, but also because they are robust methods with high resolution and precision [34,35,36].

The traditional visual inspection method is based on image processing, feature extraction, and other means to detect a tool’s surface condition. Wang et al. proposed [2] a TCM method by extracting the texture features of the tool’s surface with a camera, which simplifies the complexity of the algorithm and significantly reduces the utilization of computer resources. Zhang et al. [37] proposed a vision-based fusion method that combines a traditional visual detection method and a deep learning method. The result shows that the method can effectively detect and evaluate small defects on the spiral cutting edges with a high detection accuracy. Brili et al. [38] used infrared imaging technology to obtain the state of the tool’s wear, and combined computer vision and deep learning methods to determine the tool’s state. Ivan Kuric et al. [39] used the improved AlexNet to achieve the automatic target detection of small samples. Xian et al. [40] used a new model of YOLOv3 combined with a triple loss network to improve the feature extraction capability of neural networks and achieve high-performance surface defect detection. Ren et al. [41] proposed a network model for surface defect detection.

However, most of the abovementioned methods require huge numbers of labeled images, which are difficult to be applied in the machining industry due to its expensive labeling efforts. Moreover, compared with general TCM problems, there are some differences in the grinding process of diamond tool detection. Firstly, the defects in the cutting edge of diamond tools are tiny, so it is necessary to develop a microscale defect detection algorithm; secondly, the traditional image-processing methods often confuse image noise with impurities and the objects to be detected; and thirdly, the research work on TCM based on deep learning is mostly based on multiple sets of data and mainly on existing learning frameworks. Few algorithms have been developed specifically for the optical imaging characteristics of diamond tools.

In this paper, we propose a method for the on-machine detection of sub-microscale defects in diamond tool grinding during the manufacturing process. This method can accurately locate diamond tool defects with sub-micron resolutions, and realize small-sample learning regarding diamond tool imaging’s characteristics. Firstly, the principle of a neural network model designed for diamond defect monitoring (named DToolnet) is proposed. Then, the experiment setup as well as the design works are detailed in the next part. In the last part of the paper, the results of the experiments are illustrated and discussed.

## 2. Structure and Principle of the DToolnet

### 2.1. An Overview of DToolnet

**Figure 2:**
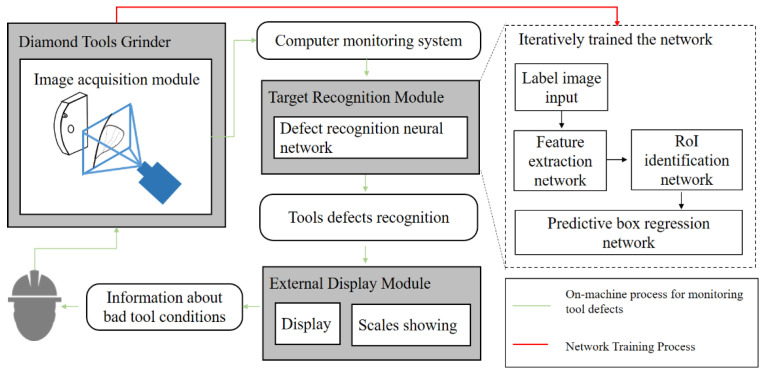
The overall process of defect identification in DToolnet.

The TCM process of diamond tool grinding is shown in Figure 2. The whole work is divided into two stages: the first is the step of establishing the feature recognition network, and the second is the step of integrating the trained network into the detection link. DToolnet’s structure can be summarized as being comprised of feature extraction, region generation, prediction box classification, and regression function modules, which are developed from Faster RCNN [42]. DToolnet uses a more innovative region proposal network (RPN) architecture compared to the previous target detection networks, and develops an anchor mechanism to link the convolutional networks and the region generation modules together. DToolnet first completes the generation of the area of interest (RoI) using the RPN module. The generated areas are then classified so that defective tools can be detected.

### 2.2. The DToolnet Feature Extraction Network

**Figure 3:**
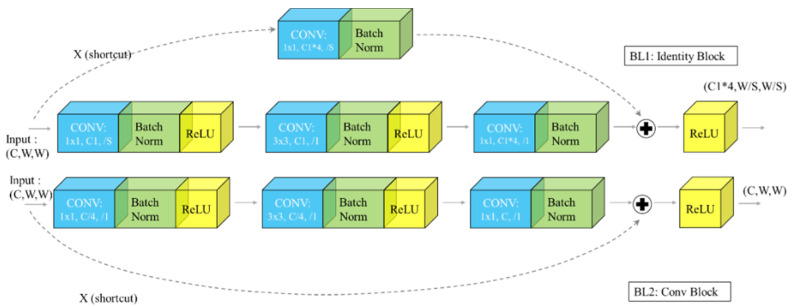
Two bottleneck structures of the DToolnet feature extraction network.

The diamond tool feature extraction network is divided into five stages, Stage 0 of which has a relatively simple structure and can be regarded as the pre-processing of input X. The last four stages are composed of blocks and have similar structures. Stage 1 contains three blocks, and the remaining three stages contain four, four, and three blocks, respectively. Blocks introduce the idea of shortcut networks, and their structure is shown in Figure 3.

DToolnet uses bottleneck structures of ResNet50 [43] to build a feature extraction network, removing the fully connected layer behind Layer 5 of ResNet and connecting with the subsequent network, which can increase the depth of the network and more effectively extract the defect features of diamond tools. Since deep convolutional networks belong to supervised learning, a large number of labeled samples are required to train the network. However, image data for diamond tool defects are not readily available, so training the network from scratch is not ideal. In order to solve the problem of overfitting brought to the network by the training of small data samples from scratch, DToolnet uses the transfer learning method to load pre-training parameters to speed up the convergence of the network and make the model easier to optimize.

**Figure 4:**
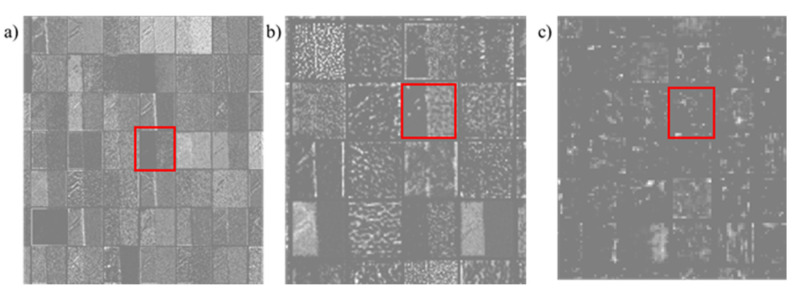
The changing process of feature-map transmission in neural networks. For the convenience of display, the feature maps of multiple channels at each stage are drawn in the form of grayscale images and spliced together. (**a**–**c**) represent the feature maps generated by Stage 1-Stage 3 of ResNet50, respectively.

**Figure 5:**
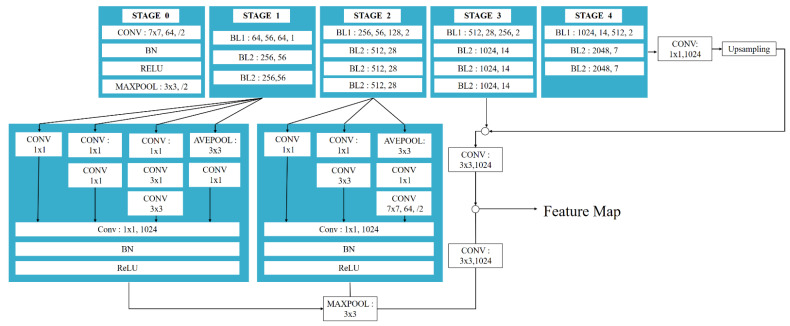
DToolnet feature extraction network structure.

Some small-scale features may disappear in the feature extraction network pass. As shown in Figure 4, one is the change of the tool image when the network is passed. The three images represent the visualization of the different channels of the feature extraction network as grayscale images. The channels enclosed in red boxes show similar feature representations. As the network deepens, the small-scale features (for example, some spots) gradually disappear. Considering that the diamond tool may produce small defects that are difficult to identify, the feature extraction network of DToolnet draws on the inception idea of GoogleNet [44] to enhance shallow-feature extraction so as to retain small features, and fuses all the features to finally output the feature map. Finally, the feature extraction network structure of DToolnet is shown in Figure 5.

### 2.3. RPN Module

**Figure 6:**
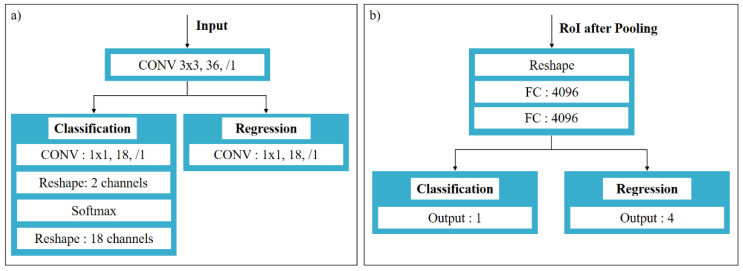
(**a**) RPN network structure; (**b**) RCNN network structure.

The work performed by DToolnet after generating the feature map through the feature extraction network is the generation of prediction boxes, which is implemented in the RPN network. Compared with the traditional method of using a sliding window to generate detection frames, DToolnet introduces a different method to directly generate nine detection frames at each position of the feature map, which are called anchors. The position of an anchor is represented by a four-dimensional vector (x,y,w,h). *x* and *y* refer to the coordinates of the anchor’s center point; *w* and *h* refer to the width and height of the anchor, respectively. The RPN network structure is divided into two routes, which are shown in Figure 6a. The role of the first line is to predict whether each anchor is a positive sample or a negative sample through Softmax classification, and the line is called the classification branch. Another line is the regression branch, which is used to represent the offset of each anchor relative to the ground truth. The ground truth is determined during the annotation of the diamond tool defect dataset, and each ground truth is a rectangular box containing defect information. Considering the identification of small-sized diamond tool defects, DToolnet uses a small scale (4,8,32) to prevent the failed detection of tiny diamond tool defects when generating anchors.

The first layer of the RPN network goes through a 3 × 3 convolutional layer for deeper feature extraction. In the classification branch, a 1 × 1 convolution kernel is used to change the number of channels of the feature map to 18 to represent the classification probability values of the positive and negative samples of the 9 anchors. In order to use Softmax for binary classification, the number of channels is reshaped to two, and only the probability values of the positive and negative anchors are retained. After Softmax classification, the number of feature map channels is reshaped to 18 again, so that the binary classification probability values of the nine anchors are obtained. The loss function of this branch adopts the cross-entropy loss function commonly used in classification tasks, as shown in Equation (1):

(1) $$L_{cls}=\overset{N}{\underset{i}{\sum }}-\mathrm{lg}\left[p_{a}^{i}\cdot p_{gt}^{i}+\left(1-p_{a}^{i}\right)\left(1-p_{gt}^{i}\right)\right]$$

**Figure 7:**
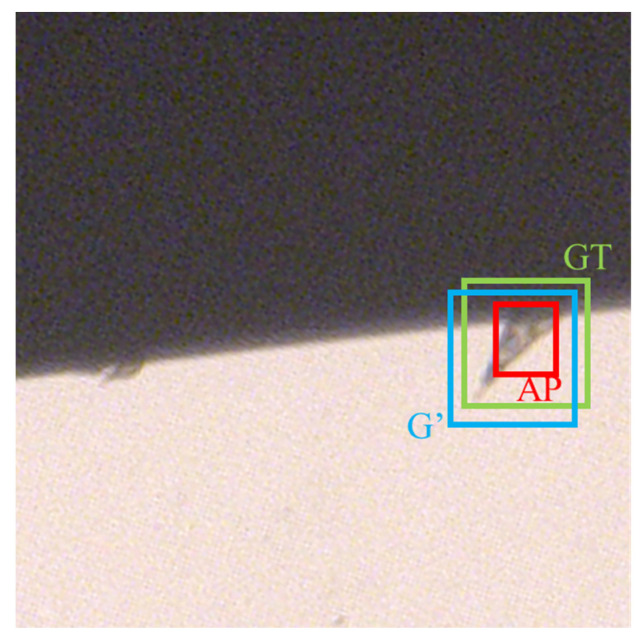
A schematic diagram of the concept in the RPN regression branch; the green box represents the ground truth, the red box represents the positive anchor, and the blue box represents the regression network result.

In the second branch, bounding box regression is performed. As shown in Figure 7, there are deviations in the positions of the positive anchors (PA) and the ground truth (GT). In order to bring the PA closer to the GT, linear mapping *F* needs to be determined. In this mapping, the original input PA becomes a regression window *G*’ close to GT’, which is shown in Equation (2).

(2) $$F\left(A_{x},A_{y},A_{w},A_{h}\right)=\left(G_{x}{}^{\prime },G_{y}{}^{\prime },G_{w}{}^{\prime },G_{h}{}^{\prime }\right)\approx \left(G_{x},G_{y},G_{w},G_{h}\right)$$

where $A_{*}$ (* represents x,y,w,h) refers to the positional parameter of PA, $G_{*}$ refers to the positional parameter of GT, and $G_{*}{}^{\prime }$ refers to the positional parameter of $G^{\prime }$.

To achieve this mapping, a simple transformation function *F* is conceived as follows:

(3) $$G_{x}^{\prime }=A_{w}\cdot d_{x}\left(A\right)+A_{x}$$

(4) $$G_{y}^{\prime }=A_{h}\cdot d_{y}\left(A\right)+A_{y}$$

(5) $$G_{w}^{\prime }=A_{w}\cdot \exp\left(d_{w}\left(A\right)\right)$$

(6) $$G_{h}^{\prime }=A_{h}\cdot \exp\left(d_{h}\left(A\right)\right)$$

In Equations (3)–(5), the four transformations of $d_{x},\;d_{y},\;d_{w}$ and $d_{h}$ need to be obtained by training the network. This transformation can be approximated as linear when PA and GT are close, so a linear regression model can be used to obtain these transformations to fine-tune the window. The linear model function can be expressed as:

(7) $$d_{*}\left(A\right)=W_{*}^{T}\cdot \phi \left(A\right)$$

where ϕ(A) refers to the feature map vector corresponding to the anchor, $W_{*}$ refers to the parameter to be learned, and $d_{*}\left(A\right)$ refers to the predicted value obtained by linear transformation. In order to minimize the difference between the predicted value and the true value, the loss function is designed as:

(8) $$Loss=\overset{N}{\underset{i}{\sum }}\left|t_{*}^{i}-W_{*}^{T}\cdot \phi \left(A^{i}\right)\right|$$

The objective of function optimization is shown in Equation (9) [42]:

(9) $${\hat{W}}_{*}={argmin}_{W_{*}}\overset{n}{\underset{i}{\sum }}\left|t_{*}^{i}-W_{*}^{T}\cdot \phi \left(A^{i}\right)\right|+\lambda \|W_{*}\|$$

In practice, DToolnet uses the smooth-L1 function to optimize the model convergence process. Therefore, the loss function calculation formula of the regression branch is shown in Equations (10) and (11) [42]:

(10) $$L_{reg}=\overset{N}{\underset{i}{\sum }}\sum_{*\in x,y,w,h}smooth_{L1}\left(t_{*}^{i}-W_{*}^{T}\cdot \phi \left(A^{i}\right)\right)$$

(11) $$smooth_{L1}\left(x\right)=\{\begin{array}{c} 0.5x^{2}\begin{array}{cc} & \;\;\;if\left|x\right|<1 \end{array}\\ \left|x\right|-0.5\begin{array}{cc} & otherwise \end{array} \end{array}$$

The final total loss function of RPN consists of two parts, as shown in Equation (12):

(12) $$L=\frac{1}{N_{cls}}L_{cls}+\lambda \frac{1}{N_{reg}}L_{reg}$$

where *λ* refers to the loss weight factor; *L* refers to the total loss function; $L_{cls}$ refers to the classification loss function; $N_{cls}$ refers to the normalized weight of classification loss; $L_{reg}$ refers to the regression loss function; and $N_{reg}$ refers to the normalized weight of regression loss.

### 2.4. The ROI Pooling Module

The subsequent fully-connected classification network requires a fixed-size input, so the feature map corresponding to the PA needs to be scaled. DToolnet utilizes the idea of Mask R-CNN [45] and introduces RoI Align to improve the quantization bias brought about by the general RoI Pooling method. In this process, the bilinear interpolation method is used to carry out the difference, and the floating-point number of each quantization is retained to avoid the loss of precision caused by rounding, to the greatest extent possible.

### 2.5. Classification and Regression Module

The classification and regression network structure is shown in Figure 6b. First, the three dimensions of the feature map obtained by RoI Pooling are expanded into one dimension. Then, the feature map is fed into a fully connected network for classification and regression. The loss function design used in the regression process is the same as that of the RPN network.

## 3. Experiment Setup

### 3.1. Hardware and Software

**Figure 8:**
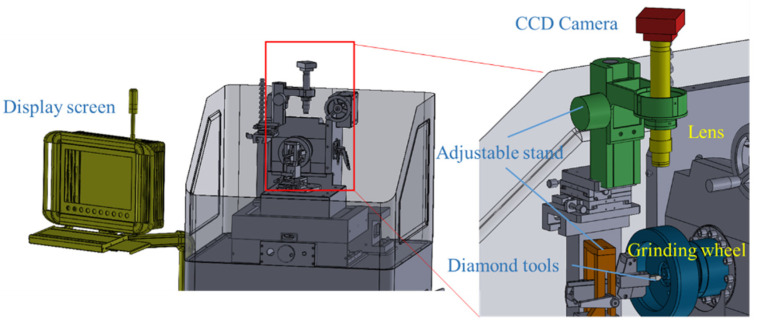
3D model of the experiment platform.

The 3D model of the relative position of the diamond tool grinder and its image acquisition system is shown in Figure 8, which shows the way to obtain the surface condition of the tool through the camera. The image system mainly consists of a camera, lens, bracket, and light source. In addition to detecting tool defects at high magnification, the imaging system should also have an auxiliary tool alignment system at low magnification. Therefore, the visual platform is designed with a 1:10 multiplication ratio to switch between different working modes. As diamond tools are usually used in the field of precision machining, it is necessary to have higher requirements for the detection of tool defects. Generally, it is required that defects of a size of 5 μm should be detected. In this case, a high-resolution industrial camera was chosen.

Considering the above problems, the component parameters of the visual imaging system are shown in Table 2.

In addition to the experiment platform, the hardware platform configuration of the training tool defect detection network is shown in Table 3. The same hardware is used for training and testing in the article. All the deep learning models used in this method are built on this platform.

### 3.2. Database

**Figure 9:**
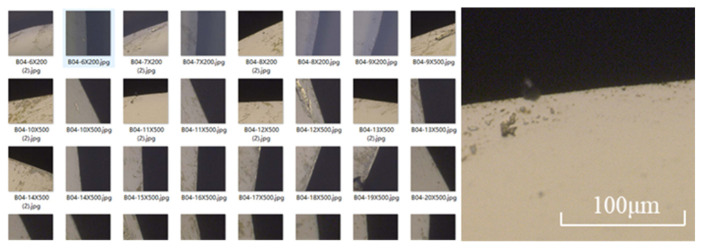
Image of diamond tool defect collected on-machine.

For training, 560 diamond tool images from two different types of cutting tools are available. For testing, 60 diamond tool images are available. All images in the dataset contain at least one defect location. Images in the dataset are cropped to 400 px × 400 px to maximize defect information. The images are taken with an optical microscope based on the platform shown in Figure 9, showing the edge of each tool. All images contain the region’s background and the undamaged tool body by default. Depending on the tool’s condition, regions depicting different tool chipping defects are present and visible as well.

### 3.3. The Experiment Material and Platform

**Figure 10:**
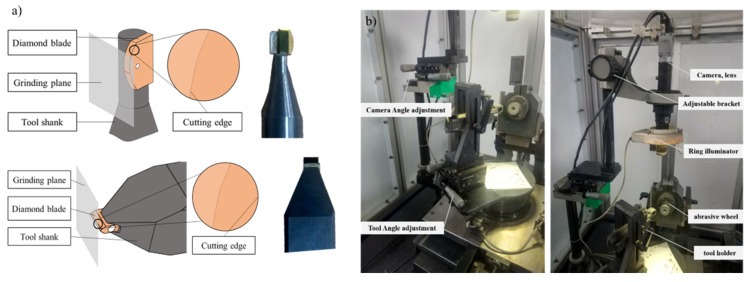
(**a**) Schematic diagram of diamond tool, (**b**) Visual inspection platform on diamond tool grinder.

There are two main forms of cutting tools processed on grinding machines, as shown in Figure 10a. The first type of shape for the tool’s cutting edge is an arc, which is mainly used for material removal in mold processing. Another kind of shape for the tool’s cutting edge is straight and with a circular outer contour, which is mainly used for material removal in the processing of molds and shells, and has a shaping function.

As shown in Figure 10b, the basic structure of diamond tools is similar to that of traditional tools. However, diamond tools are usually used in the field of ultra-precision machining; hence, the size of the tool is generally small. In this experiment, the image information of the tool’s tip fillet is captured by the lens. The amplification lens of a CMOS sensor is installed on the diamond tool grinder, so that the tool can be detected during the process of machining. Ultra-precision machining requires a tool with high surface quality, which requires that the TCM system have the ability to detect very small defects. Optical amplification can be used to obtain the cutter’s image. In this experiment, a 500X amplification lens is used to magnify the tool. The device used to acquire information about the tool image, shown in the figure, consists of an industrial digital camera with an amplification function, an annular light source, an adjusting bracket, a notebook computer, and a precision diamond tool grinder. All images used in the experiments were acquired by the vision system shown in Figure 10b.

## 4. Result and Discussion

### 4.1. Evaluation Metrics

#### 4.1.1. Loss during Training

During the training process, DToolnet updates the network weight parameters by calculating the degree of deviation between the predicted value and the label value (ground truth). The training loss and training validation loss can reflect the performance and fit of the network on the training set. As the number of training epochs increases, the loss value of the training process decreases. A neural network can be considered to be trained when the loss on the training validation set is essentially unchanged.

#### 4.1.2. Intersection over Union

**Figure 11:**
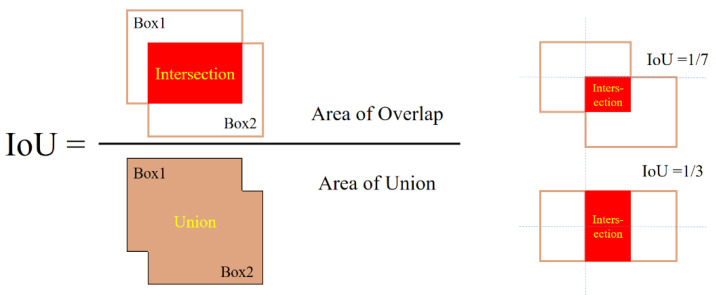
Schematic diagram of the calculation of IoU.

To evaluate the relationship between the results predicted by DToolnet and the ground truth, the article uses intersection over union (IoU) as an indicator to measure the precision of the prediction result frame. Figure 11 shows the calculation process of IoU. IoU is a value that measures the proximity of two marked boxes, and its calculation result is expressed by the ratio of the intersection area and the union area of the two marked boxes.

Feeding test images into a neural network yields a range of results. So, the recognition result of DToolnet was compared with the ground truth of the test image and the IoU value of each prediction result. IoU can indicate the precision of each prediction result. The larger the IOU, the closer the prediction result to the ground truth.

#### 4.1.3. Performance of DToolnet on the Test Set

The test set was used to evaluate the performance of the model for diamond tool defect detection after the DToolnet network training was completed.

**Figure 12:**
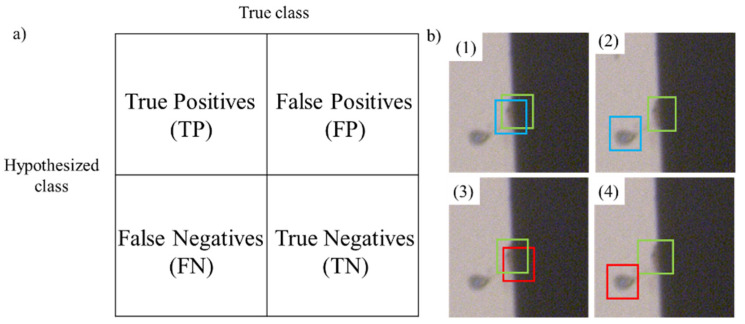
(**a**) Conceptual explanation of evaluation metric; (**b**) Case diagram of the diamond tool detection process. In these images, the green boxes represent the ground truth, the blue box represents that the algorithm recognizes the target as a positive example, and the red box represents that the algorithm recognizes the target as a negative example. (1)–(4) correspond to the four cases in (**a**) respectively.

Figure 12 illustrates several concepts related to the test results.

True Positive (TP) indicates the number of predicted results for which both the predicted results and the label values are positive samples. False Positive (FP) means the number of predicted results with positive results and negative label values. True Negative (TN) indicates the number of prediction results and the label values are negative samples. False Positive (FN) indicates the number of prediction results for which both the prediction result and the label value are negative samples. Precision, Recall, F1 measurement, and average precision (AP) are used to measure the performance of DToolnet on the test set, and they are calculated as follows:

(13) $$\mathrm{Pr}\mathrm{ecsion}=\frac{\mathrm{TP}}{\mathrm{TP}+\mathrm{FP}}\times 100\%$$

(14) Recall=TPTP+FN×100%

(15) $$\mathrm{AP}=\left(\frac{1}{n}\times \frac{\mathrm{TP}}{\mathrm{TP}+\mathrm{FP}}\right)\times 100\%$$

### 4.2. Result and Discussion

#### 4.2.1. Result of the Loss during Training

**Figure 13:**
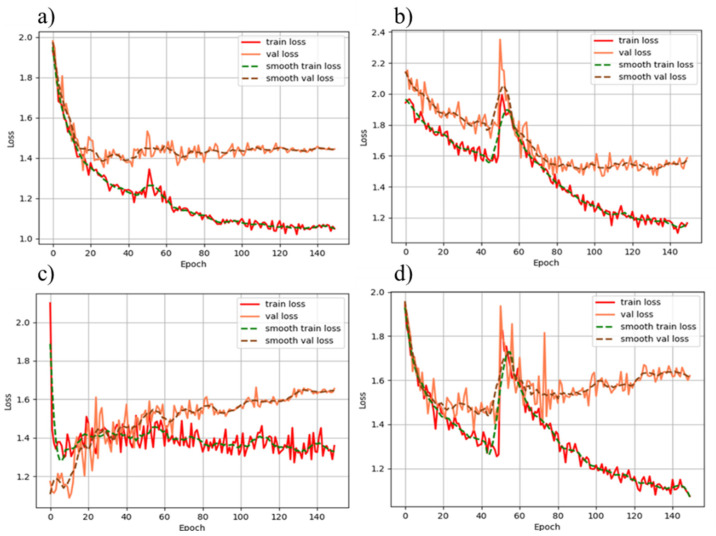
Comparison of different network training losses: (**a**) Loss when training with DToolnet; (**b**) Loss when training with a resnet50-based Faster RCNN; (**c**) Loss when training without a transfer learning network; (**d**) Loss when training with a vgg16-based Faster RCNN.

Figure 13 is a comparison of the losses of four different types of networks during training: resnet50-based [44] Faster RCNN [46], vgg16-based [47] Faster RCNN, Faster RCNN without a pre-training model process, and DToolnet. Each graph in Figure 13 contains the change in loss for each epoch of training and the change in loss of the current model for the validation set. Different from the test set, the validation set performs a loss calculation on the current network graph after each epoch of network training is completed. Figure 13a shows the training-loss relationship of DToolnet. After 90 epochs of training, the loss for the validation set basically does not change, and the basic training of the network can be considered complete at this time. At epoch 50, the small increase in loss is due to freezing the feature extraction network for the first 50 epochs of training. This can not only prevent the parameters of the feature extraction network loaded with pre-trained parameters from being destroyed, but also speeds up the training process. The other two feature extraction networks shown in Figure 13b,d produced larger fluctuations in loss values at epoch 50, and the loss for the validation set remained stable after epoch 120, indicating that their training convergence rate is lower for DToolnet. Figure 13c shows the network training process without pre-training parameters. The loss for the validation set does not tend to converge, which shows that when the dataset is relatively small, it is difficult to make the network perform well without using pre-training parameters and by increasing the number of training epochs and other parameter-tuning methods. The fact that the curve has no loss fluctuation at 50 epochs is because the process of freezing the network is not added in the early stage when the pre-training parameters are not loaded.

#### 4.2.2. Comparison of the IoU and the Mean IoU

**Figure 14:**
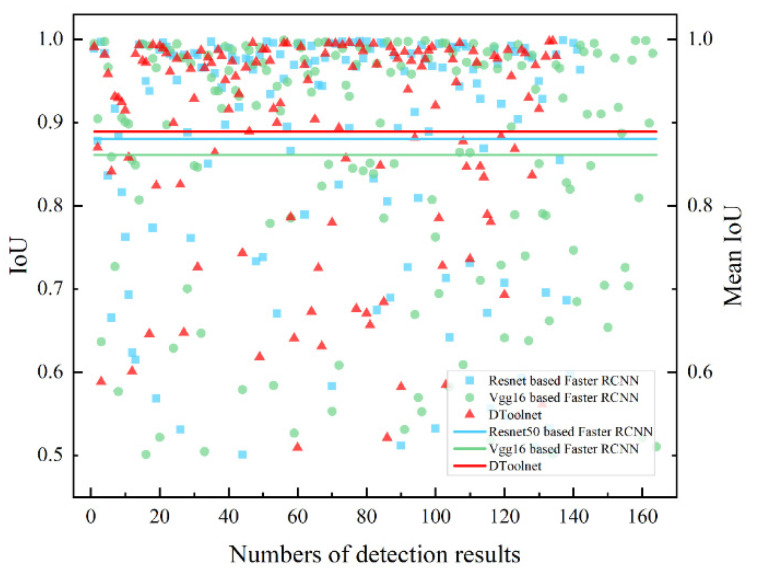
Comparison of IoU of different networks.

Figure 14 shows the IoU statistics calculated based on the prediction results produced by the three networks for the 60 test images. Each point in the figure represents the IoU value of the network output prediction box. It should be noticed that the number of predictions produced by different networks on the same test set is also different. Each test image has more than one target, and each network has a different number of target detection output prediction boxes, so the total number of prediction boxes through different networks is also different. The statistics in Figure 14 shows that the average IoU of the results predicted by DToolnet is the highest at 0.89, and the average IoU of the other two networks is 0.88 and 0.85, which proves that DToolnet has more advantages regarding the accuracy of the average prediction results.

#### 4.2.3. Comparison Results of Different Networks on the Test Set

**Figure 15:**
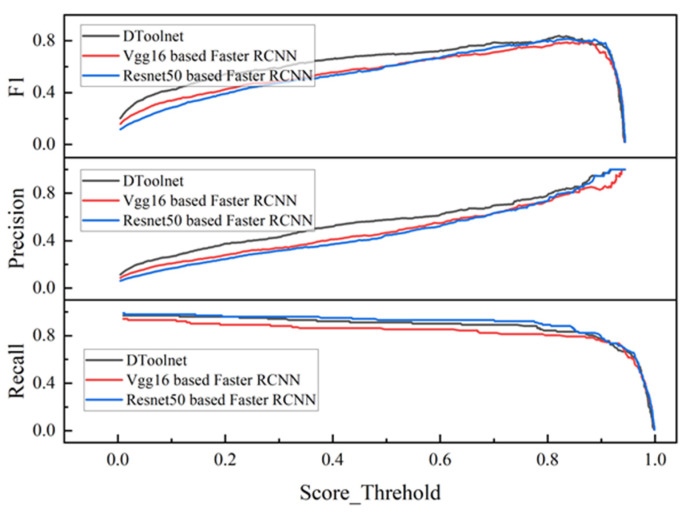
The performance of different networks on the test set.

**Figure 16:**
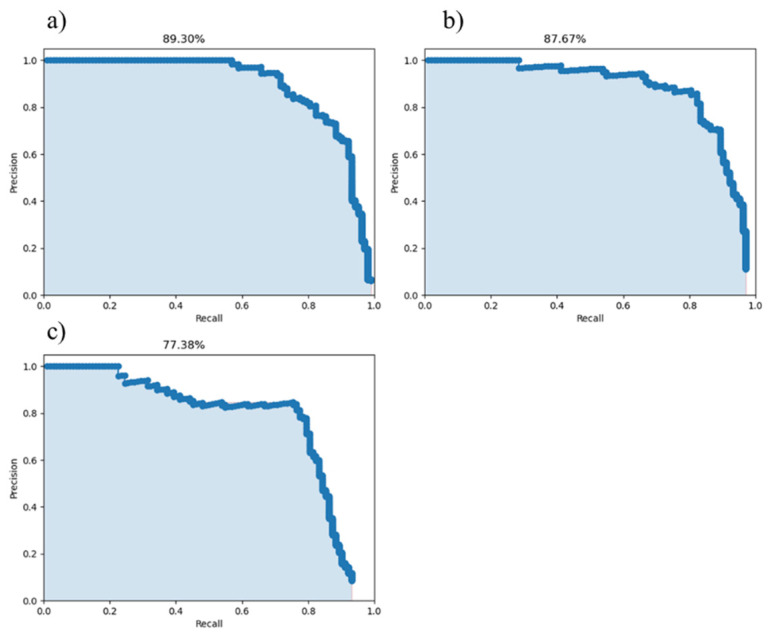
AP comparison of three networks: (**a**) AP of DToolnet; (**b**) AP of resnet50-based Faster RCNN; (**c**) AP of vgg16-based Faster RCNN.

Figure 15 shows the relationship between F1, Precision, and Recall and Score_Threhold. In the process of practical application, it is necessary to ensure that the network recognition effect is sufficiently accurate, but the Recall cannot be significantly reduced. Compared with the Precision, when the Recall value of the three networks is around 0.85, DToolnet has obvious advantages. When the Score_Threhold is set to 0.85, it can ensure that DToolnet has 90% recognition accuracy, and the recall remains above 0.7. F1 score is an indicator that combines Precision and Recall. In Figure 15, it can be seen that the F1 score of DToolnet is also higher than the other two networks, which shows that DToolnet has better overall performance. Figure 16 plots the AP curves of the three networks. DToolnet can achieve an average precision of 89.3%, which is also better than the other two network models.

## 5. Conclusions

This article aims to solve the problem of monitoring the condition of diamond tools during the process of grinding. The machine-vision-based method belongs to the direct measurement method. Compared with other indirect measurement methods, the visual method is more stable in its ability to capture the changing signals caused by tiny chippings on the cutting edge of the tool when grinding diamond tools. Compared with the general direct method, the visual method avoids the problem of efficiency loss caused by the disassembly of the diamond tool.

For the recognition of defect targets in the images collected by the vision system, this paper proposes a network structure based on the principle of deep learning, called DToolnet. Considering the problems of small target detection and small sample training in target recognition work, DToolnet has made targeted improvements. First, DToolnet enhances the extraction of small-scale targets in the feature extraction network, and fuses the feature map from the previous stage of the feature network and the final feature map for output. In this process, DToolnet combines the characteristics of the GoogleNet’s Inception and the ResNet network. Second, the target recognition network in DToolnet also reduces the size of the automatically generated anchor to help the problem of small-scale targets. The RoI Align idea in the RoI Pooling stage is used to improve the accuracy of the network regression prediction frame. Third, DToolnet draws on the idea of transfer learning and uses pre-training parameters to speed up network convergence and reduce the risk of overfitting.

After determining the solution for diamond tool inspection, the corresponding experimental hardware platform was built to obtain the data required for the experiment. Then, related evaluation metrics and comparisons between DToolnet and other networks are used to measure the performance of Dtoolnet. Through the loss performance of different networks during training, Dtoolnet shows a faster convergence speed and lower loss value under the blessing of pre-training weights. Through the comparison of the average IoU of different networks, the detection precision of DToolnet is also 4.7% higher than that of the same type of network. Finally, the performance of DToolnet on the test set is also better than other comparison networks, and an average accuracy rate of 89.3% for diamond tool defects was achieved.

However, some future work still needs to be performed. To solve the problem of small data samples, we can learn from the idea of generative adversarial network (GANs) and generate images of diamond tool defects on the basis of existing data sets. I believe this must be an interesting and worthwhile topic.

## Figures and Tables

**Table 1:** Common TCM methods.

Method		Sensors/ Instruments
Direct	Optical image	Optical sensor [10]
	Contact	Probe [11,12], magnetic gap sensor [13]
	Radiometric technique	Radioactive element [14,15]
Indirect	Cutting temperature	Thermocouple [16], infrared temperature [17]
	Surface roughness	Surface roughness measuring instrument [18,19,20]
	Ultrasonic	Ultrasonic heat generator and receiver [21]
	Vibration	Accelerometer [22], vibration sensor [23]
	Cutting force	Strain sensor [24], voltage power sensor [25]
	Power	Power sensor [26]
	Current	current sensors [27]
	Acoustic emission	Acoustic emission sensor [28]

**Table 2:** Camera and lens parameters.

Camera		Basler acA2440-75uc	
Photosensitive chip supplier	Sony	Photosensitive chip	IMX250
Shutter	Global Shutter	Chip size	2/3
Photosensitive chip type	CMOS	Photosensitive chip size	8.4 mm × 7.1 mm
Horizontal/Vertical Resolution	2448 px × 2048 px	Resolution	5 MP
Horizontal/Vertical Pixel Size	3.45 µm × 3.45 µm	Frame rate	75 fps
Color	Color	interface	USB 3.0
**Lens**		**POMEAS VP-LZL-12105**	
Working distance	77 ± 2 mm	Interface	C-Mount
Optical magnification	0.58X~7.5X		

**Table 3:** Hardware parameters used to train and test the network.

	GPU	CPU
Training platform	NVIDIA GeForce RTX 2060i	Intel (R) i7-10700
Testing platform	NVIDIA GeForce RTX 2060i	Intel(R) i7-10700

## Data Availability

Not applicable.
